# Trajectories of brain volumes in young children are associated with maternal education

**DOI:** 10.1002/hbm.26271

**Published:** 2023-03-10

**Authors:** Changbo Zhu, Yaqing Chen, Hans‐Georg Müller, Jane‐Ling Wang, Jonathan O'Muircheartaigh, Muriel Bruchhage, Sean Deoni

**Affiliations:** ^1^ Department of Applied and Computational Mathematics and Statistics University of Notre Dame South Bend Indiana USA; ^2^ Department of Statistics Rutgers University New Brunswick New Jersey USA; ^3^ Department of Statistics University of California Davis California USA; ^4^ Centre for the Developing Brain, School of Biomedical Engineering and Imaging Sciences King's College London London UK; ^5^ Department of Forensic and Neurodevelopmental Sciences King's College London London UK; ^6^ MRC Centre for Neurodevelopmental Disorders King's College London London UK; ^7^ Department of Diagnostic Imaging Rhode Island Hospital Providence Rhode Island USA; ^8^ Department of Pediatrics Warren Alpert Medical School at Brown University Providence Rhode Island USA; ^9^ Institute of Social Studies University of Stavanger Stavanger Norway; ^10^ MNCH D&T Bill & Melinda Gates Foundation Seattle Washington USA

**Keywords:** brain volumes, cerebrospinal fluid, compositional data, functional principal component analysis, grey matter, longitudinal brain development, white matter

## Abstract

Brain growth in early childhood is reflected in the evolution of proportional cerebrospinal fluid volumes (pCSF), grey matter (pGM), and white matter (pWM). We study brain development as reflected in the relative fractions of these three tissues for a cohort of 388 children that were longitudinally followed between the ages of 18 and 96 months. We introduce statistical methodology (Riemannian Principal Analysis through Conditional Expectation, RPACE) that addresses major challenges that are of general interest for the analysis of longitudinal neuroimaging data, including the sparsity of the longitudinal observations over time and the compositional structure of the relative brain volumes. Applying the RPACE methodology, we find that longitudinal growth as reflected by tissue composition differs significantly for children of mothers with higher and lower maternal education levels.

## Graphical abstract

We study brain development as reflected in the relative fractions of cerebrospinal fluid volumes (pCSF), grey matter (pGM), and white matter (pWM) for a cohort of 388 children that were longitudinally followed between the ages of 18 and 96 months. We find that longitudinal growth as reflected by tissue composition differs significantly for children of mothers with higher and lower maternal education levels.

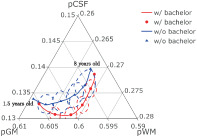

**Abbreviations**
pCSF: proportion of cerebrospinal fluid
pGM: proportion of grey matter
pWM: proportion of white matter
RPACE: Riemannian Principal Analysis through Conditional Expectation

## 1 INTRODUCTION

Longitudinal brain development through early childhood can be assessed through magnetic resonance imaging (MRI), with scans repeatedly performed for the children in a cohort at different ages. Useful features such as brain volumes reflecting brain development can then be extracted from the MRI brain images. However, the situation in many longitudinal neuroimaging studies is that for many children one may have available only very few scans and different children are usually scanned at different ages, as participants may miss scans or join the study at different ages. The analysis of such data falls in the realm of sparse longitudinal data analysis. We focus here on relative tissue volumes to quantify brain development. Focusing on relative volumes automatically adjusts for differences in total brain volumes, for example between boys and girls which are known to have different total volumes.

The most prominent distinguishable sub‐volumes of brain that can be extracted from MRI scans are labeled as cerebrospinal fluid (CSF), grey matter (GM), and white matter (WM). It is customary to adjust the observed volumes of CSF, WM, and GM by total brain volume, which leads to recordings of proportional cerebrospinal fluid volumes (pCSF), proportional grey matter (pGM), and proportional white matter (pWM) (Chen et al., 2021). These are compositional data as the fractions sum to 1 and are nonnegative (Aitchison, 1982). The use of proportional brain volumes is very common throughout neuroscience, for example, for the diagnosis of neurological diseases (Khan et al., 2021; Mito et al., 2020; Richter et al., 2021), yet systematic methodology to adjust for the compositional nature of such neuroimaging data has been at best sparsely adopted if at all. One of the goals of this article is to remedy this situation and to demonstrate how adequate compositional methods can be deployed in longitudinal studies.

Modeling and inference for longitudinal compositional data are challenging, especially when, as is the case in many neuroimaging studies, the trajectory of brain growth for each child is only observed at a few randomly located time points. Another difficulty is that the compositional space in which the proportional volumes reside is nonlinear and conventional arithmetic operations are not available, as proportions always need to remain nonnegative and add up to 1. Longitudinal compositional data analysis is therefore inherently of interest for the analysis of brain development. Although both compositional data and longitudinal data command a substantial respective literature, work on their intersections is scarce, especially for neurodevelopmental data, where the compositional structure has been largely ignored. For compositional data analysis in general, we refer to Aitchison (1982); Hadjipantelis et al. (2015); Scealy and Welsh (2011); Li (2015); Pawlowsky‐Glahn et al. (2015) among others.

To handle longitudinal data without considering the compositional nature of observations, the most commonly used approaches are based on mixed effects modeling, where one uses fixed effects for population effects and random effects for individual differences (Bernal‐Rusiel, Greve, et al., 2013; Bernal‐Rusiel, Reuter, et al., 2013; Lindstrom & Bates, 1990; Pinheiro & Bates, 2006; Sanford et al., 2018). An alternative and often preferable approach is functional data analysis Chen et al. (2021) where random effects are included in the form of functional principal component scores. An extension of mixed effects modeling to longitudinal compositional data was developed in Chen and Li (2016) with a focus on microbiome data. Since brain development trajectories follow nonlinear patterns (Bray et al., 2015; Gennatas et al., 2017; Giorgio et al., 2010; Gogtay & Thompson, 2010; Lebel et al., 2008; Lebel & Beaulieu, 2011; Tamnes et al., 2017; Yu et al., 2020), parametric approaches based on mixed effects modeling are often suboptimal due to model misspecification.

To capture nonlinear trends without a priori assumptions about the nature of the time courses as is required for parametric random effects models, we first map the compositional vectors containing pCSF, pGM, and pWM to a sphere, by applying a pointwise square root transformation (Scealy & Welsh, 2011). Once the data have been mapped to the sphere, Riemannian functional principal component analysis through conditional expectation (RPACE) (Dai et al., 2021) can be adopted for modeling longitudinal compositional data on the sphere. This utilizes the fact that the sphere is a smooth Riemannian manifold with a well‐known geometric structure. The application of RPACE makes it then possible to predict a child's entire trajectory of pCSF, pGM, and pWM over all ages. Subsequently, comparisons of trajectories of pCSF, pGM, and pWM between groups of children can be conducted conveniently using existing methods. We demonstrate this by comparing the trajectories of groups of children differentiated by maternal education, where we detected significant differences in relative brain volume development.

## 2 METHODS AND MATERIALS

### 2.1 Subject details and demographics

The brain image data used in this work were collected in the framework of the RESONANCE study, based at Brown University in Providence, RI, United States. RESONANCE is an ongoing longitudinal study of early brain and cognitive development for children from early childhood to preadolescence (Bruchhage et al., 2020). Different approaches including online advertisements, newspaper, handbill, and pediatric hospitals referrals were used to recruit children from Providence and surrounding areas. Longitudinal neurodevelopment measures such as multi‐modal MRI, cognitive and behavioral functioning and anthropometry are taken at each visit. The RESONANCE study focuses on healthy brain development and prescreening was conducted at the study enrollment to exclude children with known risk factors for developmental abnormalities. Specific exclusion criteria include: alcohol, cigarette smoking or illicit substance exposure in utero; preterm before 37 weeks gestation; weight less than 1500 g at gestation age; APGAR scores <8; complicated pregnancy and delivery; abnormalities on ultrasound; neurological disorder; and psychiatric or learning disabilities.

**FIGURE 1:**
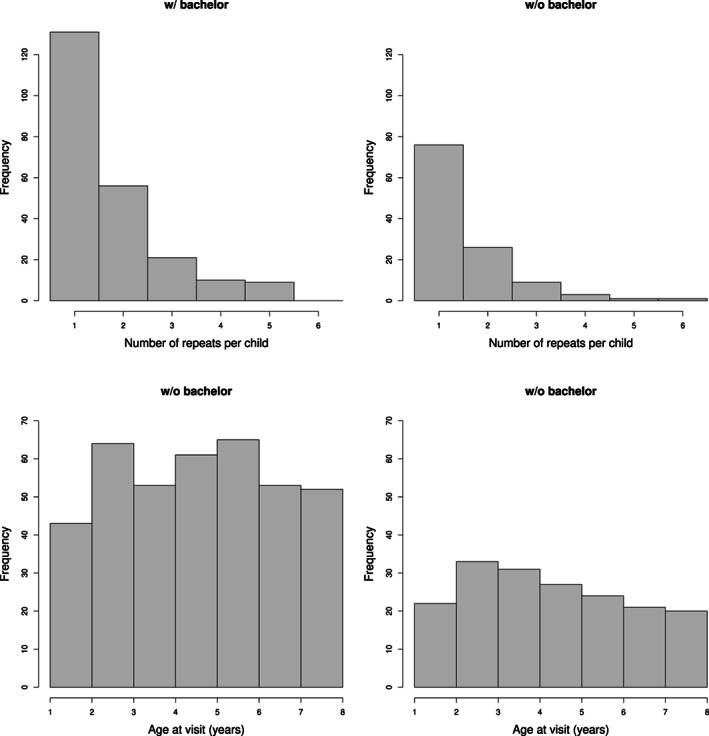
Histograms for the number of repeated measurements per child (top) and for age at visit (bottom), for the group of children whose mothers have a bachelor's degree (left) and children whose mothers do not have a bachelor's degree (right).

**FIGURE 2:**
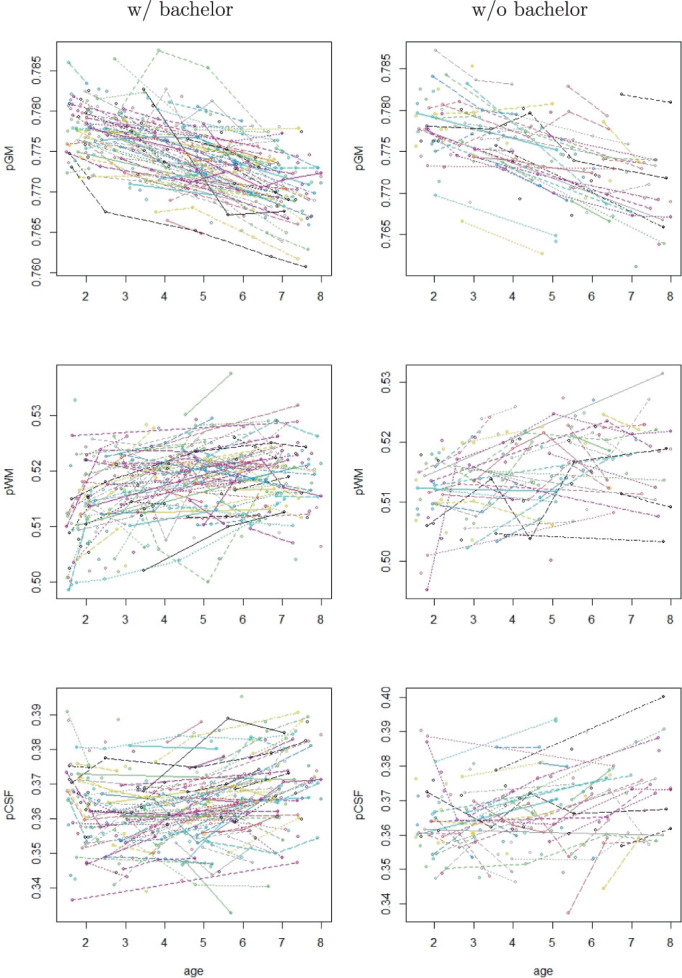
Plots of observed trajectories.

In this work, we used the data of 343 typically‐developing children from the RESONANCE cohort, where 227 children's mothers have a bachelor's degree and above, 116 children's mothers do not have a bachelor's degree and the educational information of 34 children's mothers are missing or unknown. The observed trajectories of pCSF, pGM, and pWM are shown in Figure 2, illustrating the longitudinal nature of the data. Participant demographics are summarized in Figure 1 using histograms, and Table 1 using frequency tables. There are in total 621 repeats, of which 391, 178, and 52 repeats are for children of mothers with a bachelor's degree or above, without bachelor's degree, and of missing or unknown educational information, respectively (Figure 2).

**TABLE 1:** Demographics in frequency tables.

(a) Frequency table for number of repeats per child	Frequency	
Number of repeats/child	w/bachelor	w/o bachelor
1	131	76
2	56	26
3	21	9
4	10	3
5	9	1
6	0	1

(b) Frequency table for age at visit	Frequency	
Age at visit	w/bachelor	w/o bachelor
1≤age<2	43	22
2≤age<3	64	33
3≤age<4	53	31
4≤age<5	61	27
5≤age<6	65	24
6≤age<7	53	21
7≤age≤8	52	20

### 2.2 Ethics statement

Research ethics oversight was provided by the host institutions, including the Brown University and Lifespan institutional review boards. For all children, written informed consent was obtained from their parents or legal guardians.

### 2.3 MRI acquisition and analysis

**FIGURE 3:**
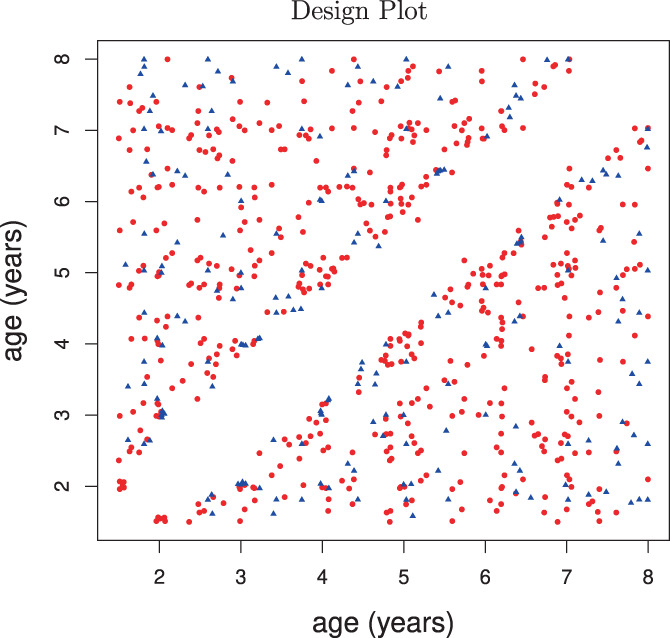
Design plot of all paired ages where observations are recorded. If a child has two measurements, the coordinates of the point in the scatterplot correspond to the ages at which the two measurements were made. If a child has one measurement, it does not produce a point in the scatterplot. If a child has three or more measurements, then each possible pair of measurements is reflected as a separate point in the plot, where the coordinates are the two ages at which the measurements are made. The plot indicates that our method is applicable for these data. Red points correspond to children with mothers who have a bachelor's degree and blue points indicate children whose mothers do not have any bachelor's degree, indicating no major design differences between these two groups.

Children were scanned during natural sleep, or when watching a favorite video. The MRI acquisition has been described in Chen et al. (2021). In brief, for each child and imaging session, a T1‐weighted anatomical inversion‐recovery spoiled gradient‐recalled echo sequence (IR‐SPGR) was acquired using a 3 T Siemens Trio scanner (with a repetition time 16 ms, echo time 6.9 ms, inversion time 950 ms and a flip angle of 15 degrees). The acquisition matrix and field of view were varied according to child head size in order to maintain a constant voxel volume and spatial resolution across all ages as in Deoni et al. (2015). To segment the regions of interest (grey matter, white matter and CSF) from a standard space, each anatomical T1‐weighted image was aligned using a multistep registration procedure (Chen et al., 2021; O'Muircheartaigh et al., 2014). This included a registration of the raw T1‐weighted image to an age‐specific template, and an additional registration from the age specific template to a study specific template (Deoni et al., 2008). Using these registrations, a grey, white and CSF segmentation in standard space was projected back to native space. This allowed the calculation of tissue volumes for native space brains. The estimated WM, GM, and CSF volumes are divided by their sum to calculate the proportional fraction (i.e., pWM = WM/[WM + GM + CSF]). The design plot (Carroll et al., 2021; Yao et al., 2005) in Figure 3 illustrates all pairwise measurements with age differentiated by maternal education level. Most of the children have only one measurement (Figure 1).

### 2.4 Statistical methods

Joint modeling and inference for the growth curve of proportions of pCSF, pGM, and pWM is challenging due to the longitudinal and compositional nature of the data. Longitudinal designs where the proportions are sparsely observed and at different ages for each child are quite common in neuroimaging data collections. The compositional structure of the data comes from the fact that each observed vector of pCSF, pGM, and pWM sums to 1, which cannot be ignored in an efficient statistical analysis.

#### 2.4.1 RPACE modeling

The Riemannian Functional Principal Analysis through Conditional Expectation (RPACE) approach is a dimension reduction and imputation method for longitudinal repeated measurements that assume values on a smooth Riemannian manifold (Dai et al., 2021). RPACE modeling is an extension of principal components analysis through conditional expectation (PACE) for longitudinal and functional data when observations lie in Euclidean space (Carroll et al., 2021; He et al., 2018; Wang et al., 2016). The main idea of RPACE is to transfer the manifold values to a linear tangent space, in which the classical PACE method (Yao et al., 2005) can be applied, and then to project the resulting values back to the manifold.

Through RPACE modeling, the longitudinal observations on the manifold can be summarized using scalar valued Riemannian functional principal component scores. To study brain development during the period of early childhood, longitudinal observations of proportional cerebrospinal fluid (pCSF), grey matter (pGM), and white matter (pWM) are collected. Then, for each child, we map each observed vector of pCSF, pGW, and pWM to the two‐dimensional sphere $S^{2}$ using the pointwise square root transformation. The sphere is a smooth manifold with a well‐studied geometric structure so that it is convenient to apply the RPACE modeling. An implementation of RPACE is available in an R package RFPCA on GitHub at https://github.com/CrossD/RFPCA.

A more technical description of RPACE is as follows. Let ℳ be a smooth, connected and geodesically complete Riemannian manifold with intrinsic distance $d_{ℳ}$ and $X\left(t\right)$, t∈T, be a ℳ‐valued stochastic process supported on the time index set T. Then the Fréchet mean function $\mu \left(t\right)$ is defined as $\mu \left(t\right)={\text{argmin}}_{p\in ℳ}E\left[d_{ℳ}^{2}(p,X\left(t\right))\right]$. Denote the tangent space attached to a point p on ℳ by $T_{p}ℳ$. For any $v\in T_{p}ℳ$, there exists a unique geodesic $\gamma_{v}:\left[0,1\right]\to ℳ$ such that $\gamma_{v}\left(0\right)=p$ and $\gamma_{v^{\prime }}\left(0\right)=v$. The Riemannian exponential map, $\mathrm{Exp}:T_{p}ℳ\to ℳ$, is defined as $\mathrm{Exp}\left(v\right)=\gamma_{v}\left(1\right)$, and the Riemannian logarithm map, $\mathrm{Log}:ℳ\to T_{p}ℳ$, is the inverse of Riemannian exponential map. For further details, we refer to Lee (2018).

By applying the Riemannian logarithm map, RPACE maps the ℳ‐valued process to the corresponding vector valued process as $L\left(t\right)={\mathrm{Log}}_{\mu \left(t\right)}X\left(t\right)$. The covariance function of $L\left(t\right)$ can be defined as $\Gamma \left(s,t\right)=E\left[L\left(s\right)L{\left(t\right)}^{T}\right],s,t\in T$, with eigendecomposition $\Gamma \left(s,t\right)=\sum_{k=1}^{\infty }\lambda_{k}\phi_{k}\left(s\right)\phi_{k}{\left(t\right)}^{T},$ where the eigenfunctions $\left\{\phi_{k}:k=1,2,\cdots ,\infty \right\}$ are orthonormal and the eigenvalues $\left\{\lambda_{k}:k=1,2,\cdots ,\infty \right\}$ satisfy conditions $\lambda_{k}\geq 0$ for any k, $\lambda_{1}\geq \lambda_{2}\geq \cdots$ and $\sum_{k=1}^{\infty }\lambda_{k}<\infty$. This leads to the Karhunen–Loève representation

$$L\left(t\right)=\sum_{k=1}^{\infty }\xi_{k}\phi_{k}\left(t\right),\;\xi_{k}=\int_{T}L{\left(t\right)}^{T}\phi_{k}\left(t\right)\mathrm{dt},$$

where $\xi_{k}$ are the uncorrelated Riemannian functional principal component scores such that $E\left[\xi_{k}\right]=0$ and $E\left[\xi_{k}^{2}\right]=\lambda_{k}$. The RPACE modeling is finalized by mapping the truncated version back to ℳ via the exponential map

$$X\left(t\right)\approx {\mathrm{Exp}}_{\mu \left(t\right)}\left(\sum_{k=1}^{K}\xi_{k}\phi_{k}\left(t\right)\right),$$

for a given integer K.

To fit RPACE for longitudinal data, consider a sample $\left\{X_{i}:i=1,2,\ldots ,n\right\}$ of an ℳ‐valued Riemannian process X and assume that each realized trajectory $X_{i}$ is only observed at random times $T_{i1},T_{i2},\ldots ,T_{{\mathrm{im}}_{i}}\in T$. Furthermore, the measurements are assumed to be corrupted by intrinsic errors so that one observes $Y_{\mathrm{ij}}={\mathrm{Exp}}_{\mu \left(T_{\mathrm{ij}}\right)}\left(L_{\mathrm{ij}}\right)\;\text{where}\;L_{\mathrm{ij}}=L_{i}\left(T_{\mathrm{ij}}\right)+\epsilon_{\mathrm{ij}}.$ Based on the observations $\left\{Y_{\mathrm{ij}}:i=1,2,\ldots ,n;j=1,2,\ldots ,m_{i}\right\}$, the mean function μ and the eigenfunctions $\left\{\phi_{k}:k=1,2,\ldots ,K\right\}$ can be consistently estimated and for each process $X_{i}$, the corresponding principal scores $\left\{\xi_{\mathrm{ik}}:k=1,2,\ldots ,K\right\}$ are estimated using the best linear unbiased predictions. Denoting these estimates by $\hat{\mu },{\hat{\phi }}_{k}$ and ${\hat{\xi }}_{\mathrm{ik}}$, we approximate the trajectory $X_{i}$ by

$$X_{i}\left(t\right)\approx {\mathrm{Exp}}_{\hat{\mu }\left(t\right)}\left(\sum_{k=1}^{K}{\hat{\xi }}_{k}{\hat{\phi }}_{k}\left(t\right)\right).$$

The randomness in each process $X_{i}$ is captured by the corresponding principal scores $\left\{{\hat{\xi }}_{\mathrm{ik}}:k=1,2,\ldots ,K\right\}$ and further statistical analysis can be conducted based the vector‐valued sample $\left\{{\hat{\xi }}_{\mathrm{ik}}:i=1,2,\ldots ,n;k=1,2,\ldots ,K\right\}$. By default, the R package RPACE works on a grid of 51 points that are equally spaced over the observed domain, that is, all estimations and computations are done on this grid, including mean and eigenfunctions. The default kernel of RPACE is the Epanechnikov kernel for both mean and covariance. We note that RPACE handles the estimation of densely and sparsely sampled data differently, as the handling of sparse data is computationally more expensive. Other relevant input arguments of RPACE from a computational perspective include whether data contain noise and the number of eigenfunctions to include in the estimation step.

#### 2.4.2 Connecting brain volumes with RPACE

Our goal is to understand the association between brain shape growth as represented by relative brain volumes and predictors such as sex and maternal education. A straightforward approach is to divide the children into groups differentiated by sex or maternal education and then to compare the trajectories of pCSF, pGM, and pWM. Unfortunately, this naive approach is not applicable, as each child's trajectory is only observed at a few time points and each observation, that is, a vector of pCSF, pGM, pWM, is a compositional data point.

**FIGURE 4:**
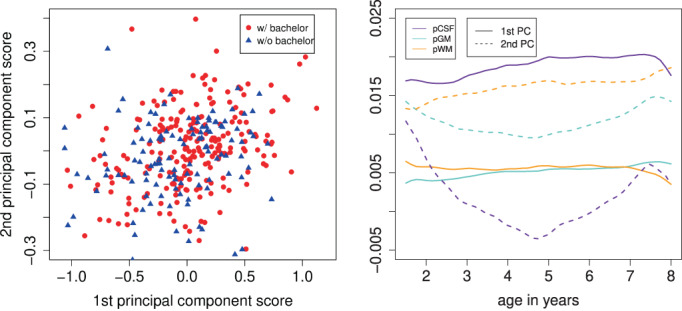
Functional principal component analysis with first and second principal component scores (left) and estimated eigenfunctions (right). Note that the eigenfunctions here are trivariate vector functions.

To address the compositional constraint, we apply to each observation of the 3‐vector pCSF, pGM, pWM a pointwise square transformation, that is, we form $(\sqrt{\text{pCSF}}$, $\sqrt{\mathrm{GM}}$, $\sqrt{\mathrm{pWM}})$, to map the three‐dimensional vector of brain volumes to the two‐dimensional sphere $S^{2}$ and then apply the RPACE approach. In other words, all available longitudinal compositional measurements are mapped to a sphere and then fed into the RPACE model to reconstruct the predicted complete trajectory of pCSF, pGM, and pWM for each child and each age. Since the first two eigenfunctions explain more than 90% of the total variation, we use just the first two Riemannian functional principal components to approximate the trajectories. Each child's trajectory is then summarized by the corresponding first two principal component scores. We then conduct statistical tests by using the first two scores, aiming to compare the trajectories of the relative volumes which are viewed as trajectories on the sphere for different groups of children. The score and eigenfunction plots are provided in Figure 4.

To assess the performance of RPACE, we compared it with fitting linear mixed effect models (LMM). Specifically, we fitted a LMM model for each of the raw brain volumes CSF, GM, and WM separately, using the standard LMM

$$\begin{array}{l} {\mathrm{CSF}}_{i,j}=a_{0}+a_{1}t_{j}+b_{i}+\epsilon_{i,j},{\mathrm{GM}}_{i,j}=c_{0}+c_{1}t_{j}+d_{i}+\varepsilon_{i,j},\\ {\mathrm{WM}}_{i,j}=h_{0}+h_{1}t_{j}+f_{i}+e_{i,j}, \end{array}$$

where ${\mathrm{CSF}}_{i,j}$, ${\mathrm{GM}}_{i,j}$, ${\mathrm{WM}}_{i,j}$ are the brain volumes for subject i at age $t_{j}$; $b_{i},d_{i},f_{i}$ are random intercepts with mean zero and finite variance; $\left\{\epsilon_{i,j},\varepsilon_{i,j},e_{i,j}\right\}$ are mutually independent mean zero finite variance random errors; $a_{0},a_{1},c_{0},c_{1},h_{0},h_{1}$ are non‐random parameters. Here, adding random slopes would result in identifiability issues due to insufficient data and a model that includes these is not identifiable and does not converge.

An interesting prediction problem is to predict the brain volume proportions of a child at a future time given past measurements. To mimic this situation, the proposed RPACE model and a standard LMM were fitted to data with the latest brain volume proportions of a selected child dropped. We then used the fitted models to predict the dropped proportions, so this is an out‐of‐sample prediction. The prediction error is measured in terms of the Fisher‐Rao distance between predicted proportions and observed proportions. We repeated this procedure for each participant with at least three scans and report the average Fisher‐Rao distance scaled by the maximum of the pairwise Fisher‐Rao distances between all observed brain volume proportions. RPACE and LMM achieved scaled prediction errors of 0.093 and 0.122, respectively. Thus the prediction error of LMM is substantially larger, which supports the application of RPACE for brain compositional data.

#### 2.4.3 Energy distance

Energy distance (ED) is a metric that quantifies the difference between two given distributions (Chakraborty & Zhang, 2021; Székely & Rizzo, 2004; Zhu & Shao, 2021). For two vector‐valued random variables $V,W\in {\mathbb{R}}^{p}$ with distributions F and G respectively, energy distance is given by

$$\mathrm{ED}\left(F,G\right)=2E\left[\left\|V-W\right\|\right]-E\left[\left\|V-V^{\prime }\right\|\right]-E\left[\left\|W-W^{\prime }\right\|\right],$$

where ‖⋅‖ is the Euclidean distance and $\left(V^{\prime },W^{\prime }\right)$ are independent copies of $\left(V,W\right)$. Energy distance has the properties that

$$\mathrm{ED}\left(F,G\right)\geq 0\;\text{and}\;\mathrm{ED}\left(F,G\right)=0\Leftrightarrow F=G.$$

To test the null hypothesis F=G, the test based on energy distance can be conveniently implemented via permutations. The distributional differences between two populations include but are by no means limited to the differences in mean and covariance structures. Here we are interested in evaluating the distributional differences in brain growth for children with different biological sex or socioeconomic status. This method has been implemented in the R package energy (Rizzo & Szekely, 2021).

#### 2.4.4 Ternary plots

A ternary plot is a graphical technique for representing three variables that sum to a constant. It has a triangular shape, where each tip of the triangle represents one of the variables. Any observation of the three variables can be represented as a point within the triangle and the closer a point is to one of the tips, the higher the proportion of the variable corresponding to this tip is; see Murrell (2005) for further details.

### 2.5 Data and code availability statement

Deidentified data are freely available upon request. Access to identifiable information will require a formal data sharing agreement and appropriate ethical approval. Requests for data should be submitted to Sean Deoni (sdeoni@mac.com). We use the R package RFPCA for implementation of RPACE, which is publicly available on github at https://github.com/CrossD/RFPCA, and also the R package energy (Rizzo & Szekely, 2021) for the energy test.

## 3 RESULTS

We first compare the trajectories of pCSF, pGM, and pWM between boys and girls. Here, we are interested to test for differences between not only the mean and covariance structure but also the underlying distributions. Thus, the null hypothesis is that the two groups of trajectories have the same underlying distributions. To implement this test, we applied energy distance based tests to the corresponding two groups of principal component scores. Only the top two scores were used in the analysis and no significant differences were found (p‐value: .40). Thus, there is no evidence that the brain growth trajectories between boys and girls are substantially different.

**FIGURE 5:**
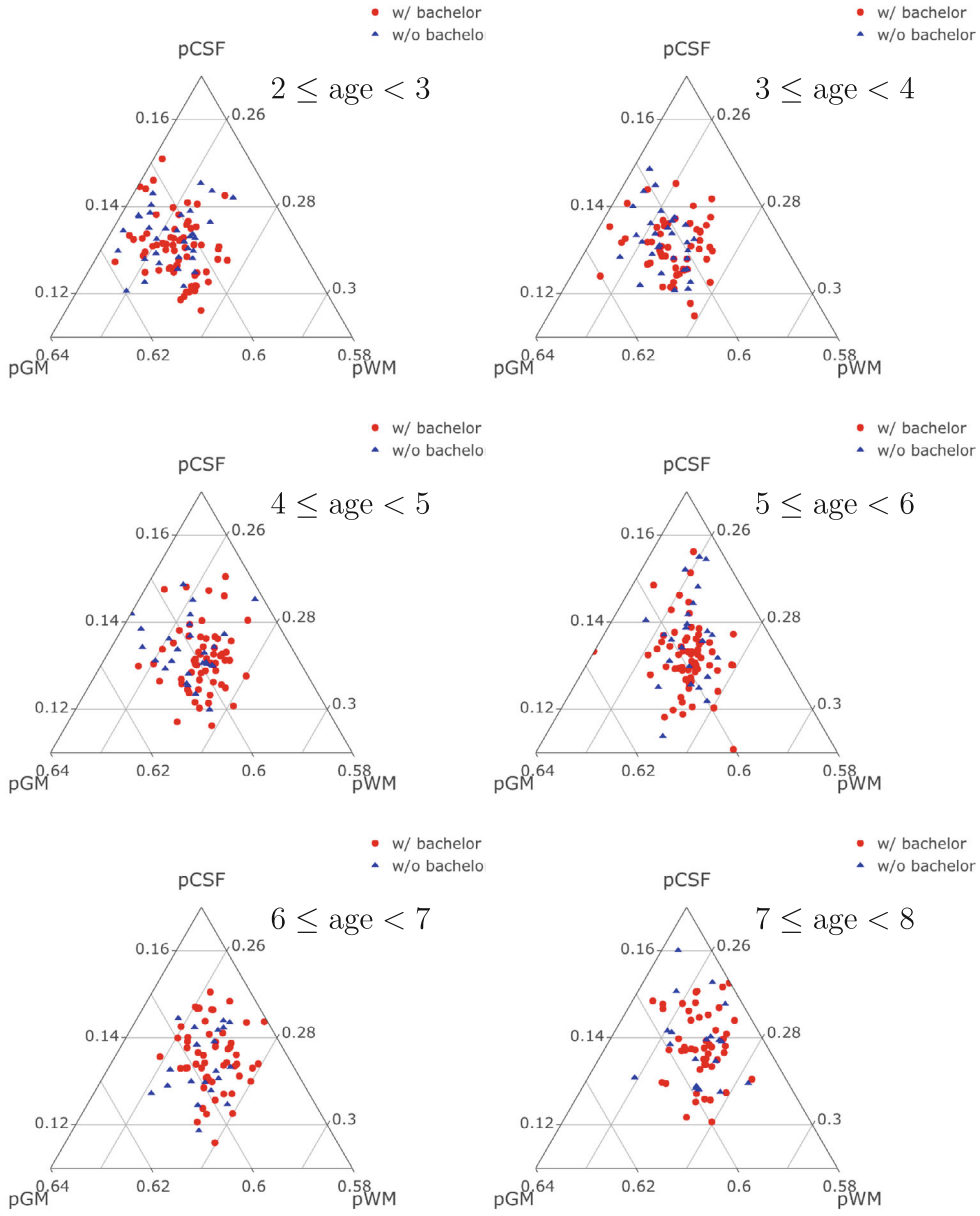
Ternary plots of pGM, pWM, and pCSF for different age groups.

Next, we compare the brain growth trajectories of children whose mothers have or do not have a bachelor's degree. The ternary plots of the compositional observations across different age ranges are shown in Figure 5. We applied the energy distance based permutation test with 9999 permutations for the first two principal components, which yielded a p‐value .033. This provides some indication that the generating distributions of the brain growth trajectories as reflected in relative volumes are different between children whose mothers do or do not have a bachelor's degree. This also motivates to apply the RPACE modeling separately for the two groups, as follows.

**FIGURE 6:**
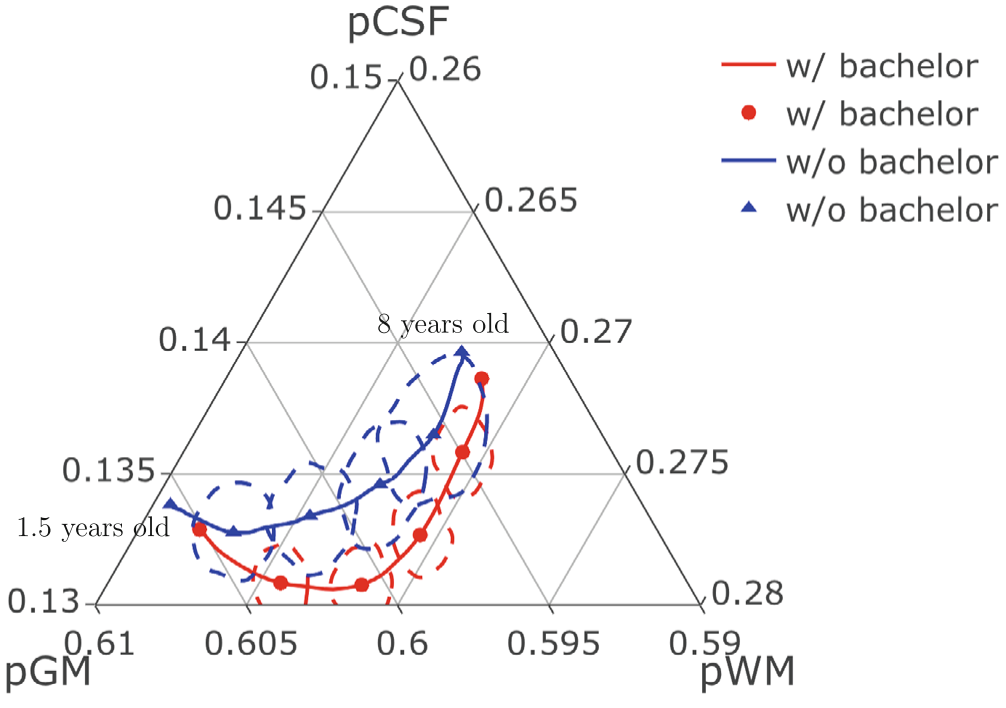
Ternary plot of mean proportions. The highlighted points on the mean trajectories (circles for mothers w/bachelor and triangles for mothers w/o bachelor) correspond to the mean values at ages 2.8, 4.1, 5.4, and 6.7. Also shown are 95% confidence regions at each of these four ages (regions within the dotted curves).

Applying RPACE modeling separately for the group of children whose mothers have a bachelor's degree and the group of children whose mothers do not, the estimated mean functions of pCSF, pGM, pWM are shown in Figure 6 in the form of ternary plots. On average, the proportion of white matter is found to be larger for the children whose mothers have a bachelor's degree. Figure 6 also indicates that the proportion of white matter increases as children grow, but the proportion increases faster for the group of children whose mothers have a bachelor's degree, especially in the age range 1.5 to 4.5 years. In addition, 95% confidence regions are shown by dashed lines for pre‐selected ages 2.8, 4.1, 5.4, and 6.7 for each of the mean functions in Figure 6. The confidence regions are constructed from the bivariate densities of 400 bootstrap samples at each age, where the densities are estimated using kernel density estimation by treating the points on ternary plot as points on ${\mathbb{R}}^{2}$ and anchoring the 95% regions on a contour of the density estimates. The confidences regions indicate that specific disparities between the two group occur around age 4.1.

**FIGURE 7:**
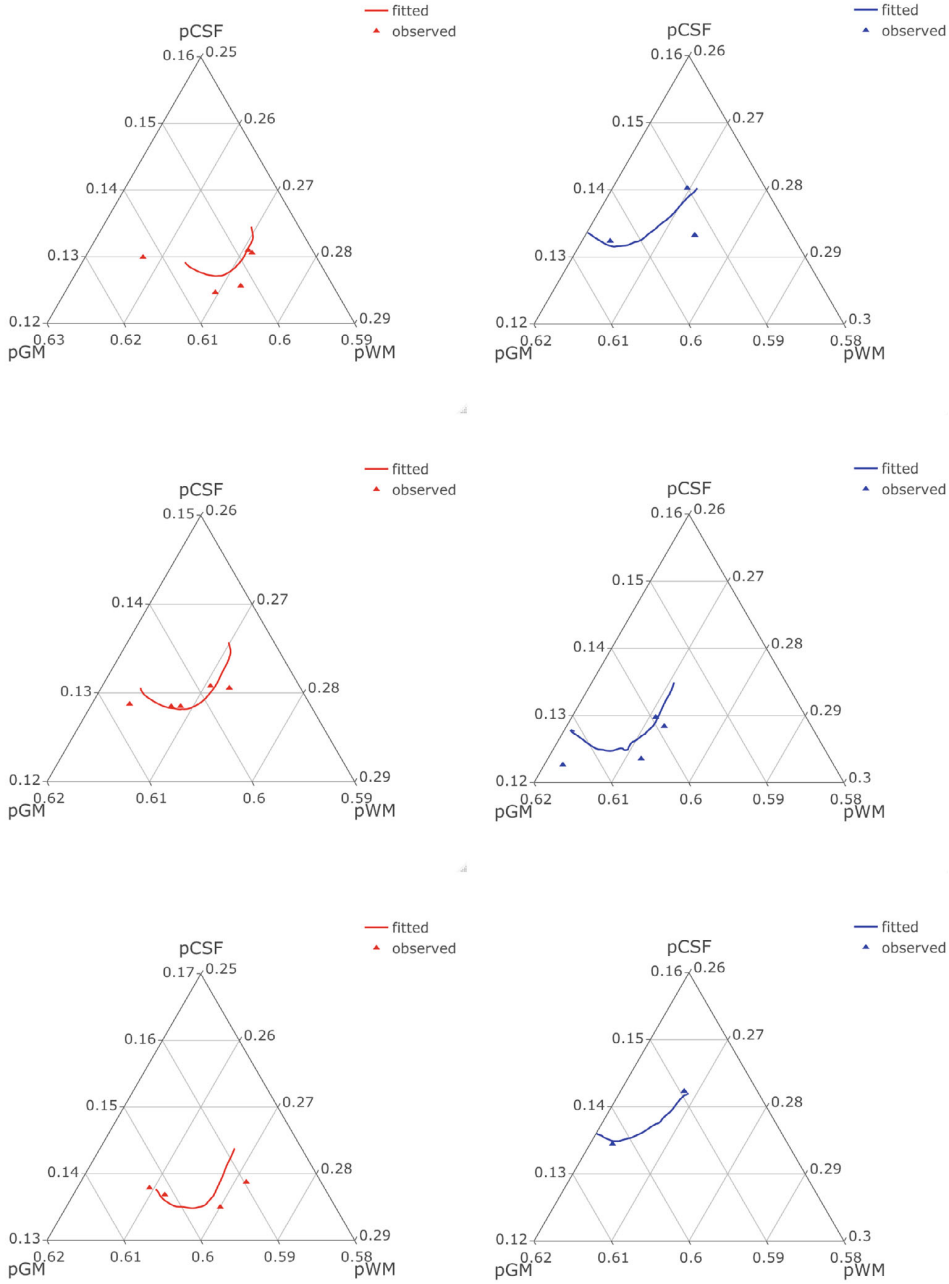
Ternary plots of reconstructed trajectories of pGM, pWM, and pCSF for a few individuals, in red for mothers w/bachelor, blue w/o bachelor. The observations are shown as triangles.

In addition, RPACE modeling makes it possible to reconstruct the predicted individual brain growth trajectories. Figure 7 shows the reconstructed trajectories of pCSF, pGM and pWM for several children with more than two observations. Trajectories of children whose mothers have a bachelor's degree are shown in red, and all other trajectories in blue.

## 4 DISCUSSION

In this work, we demonstrate the usefulness of RPACE to estimate and reconstruct brain growth trajectories in children in the presence of sparse sampling in time. Specifically, we quantified brain growth by using volume proportions of CSF, GM, and WM, and RPACE proved suitable for modeling the resulting brain volume data.

This addresses two challenges: First, proportional volumes do not lie in a vector space and thus ordinary arithmetic operations such as addition and subtraction are not available; second, for most neuroimaging data, only very few measurements are collected over time for each subject and different subjects may be measured at different time points. To tackle these two issues, we map the proportional volumes to a sphere by taking a pointwise square root transformation and utilize the resulting geometrical structure.

We note that the transformations of brain volumes are constrained to be in the first octant of the sphere, more generally in the first orthant that corresponds to the positive segment, that is, the segment of the sphere with positive coordinates. It is possible that the predicted volumes of RPACE lie outside this segment. This is more likely to happen if there are data points in the training set lying on or near the boundary of the positive segment. For the brain volume proportions used in this article, there are no points near the boundary, the observed proportions are strictly away from 0 and therefore this issue does not arise. But even if the training data include points on or near the boundary, this issue can be easily addressed by projecting any prediction that lies outside the positive segment back to the positive segment, that is, finding the boundary point of the segment that is closest in the Fisher‐Rao (or geodesic) metric to the point that is outside of the segment, see Zhu and Müller (2022) for more discussion on these projections.

More generally, the RPACE approach is suitable for modeling any longitudinal data that lie on a smooth manifold and is not limited to data on spheres. It employs local linearization by invoking the tangent space of the smooth Riemannian manifold that is anchored at the Fréchet mean of the data.

We then compared brain growth trajectories between different groups of children. There are many factors that can affect a child's brain development such as genetic factors or surrounding environment. In this article, we focus on investigating the role of biological sex and maternal education. While we found no evidence that brain growth trajectories differ between boys and girls, we observed significant differences in relative brain volumes for children whose mothers do and those who do not have a bachelor's degree (Figure 5). Specifically, ternary plots of estimated mean functions of pCSF, pGM, pWM demonstrate that the proportion of white matter was larger for the children whose mothers have a bachelor's degree (Figure 6). While proportional white matter increased with age for all children, it increased faster for the group whose mothers had a bachelor's degree, especially in the age range 1.5 to 4.5 years (Figure 6).

Structural brain development follows a nonlinear trajectory at both whole‐brain and regional brain structure levels, with gray matter volume increasing rapidly during infancy, peaking within the first 3 years of life (Matsuzawa et al., 2001) and gradually decreasing thereafter. In contrast, white matter volume increases much longer, spanning childhood and early adolescence (Barnea‐Goraly et al., 2005; Blakemore & Choudhury, 2006; Dai et al., 2019) before decreasing in older adulthood.

Healthy brain development has been identified as a key predictor of current and future cognitive development (for a review, see Gilmore et al., 2018), and therefore it is important to identify factors that could influence early brain development. Indeed, childhood and adolescence are sensitive developmental periods of dynamic behavioral, cognitive and emotional development, paralleled by significant changes in white matter micro‐ and macrostructure (Paus et al., 1999; Pfefferbaum et al., 1994; Reiss et al., 1996; Schmithorst et al., 2002), which are thought to be potential critical factors in supporting optimal cognitive, behavioral, and emotional development. Interestingly, children who suffered early neglect showed significant differences in white matter integration and cognitive function when compared to those raised in typical environments (Hanson et al., 2013), potentially indicating high sensitivity of this structure to environmental factors influencing early brain development.

Maternal education has previously been shown to strongly correlate with child physical and cognitive health and development (Bradley & Corwyn, 2002; Chen et al., 2021; Desai & Alva, 1998; Dollaghan et al., 1999; Yakovlev, 1967). In low income families, maternal education has been shown to explain language disparities emerging during early childhood (Justice et al., 2020) and a recent study (Dai et al., 2019) found the effect of maternal education to increase with age when investigating longitudinal associations between white matter maturation and cognitive development across early childhood. Specifically, this effect became significant by 1.5–2 years of age and an additional positive association between maternal education and cognition was shown, similarly increasing with child age. This aligns with our findings of steep early increases in the discrepancy of pWM mean proportions between children with low maternal (no bachelor's degree) and high maternal (with bachelor's degree) education (see Figure 5). Furthermore, it could indicate an early “window of opportunity” for 1.5–2 years of age, during which interventions could be most effective at minimizing later disparities (Campbell & Ramey, 1994).

## 5 CONCLUSIONS

The RPACE approach can handle longitudinal compositional data as they are observed for growth trajectories in terms of volume proportions of CSF, GM, and WM. Application of this method led to the detection of significant differences in the underlying distributions of brain growth trajectories between children based on maternal education, while no significant difference was found between boys and girls.

## CONFLICT OF INTEREST

The authors declare no relevant conflicts of interest.

## Data Availability

Deidentified data are freely available upon request. Access to identifiable information will require a formal data sharing agreement and appropriate ethical approval. Requests for data should be submitted to Sean Deoni (sdeoni@mac.com).
